# *De novo* transcriptome sequencing in a songbird, the dark-eyed junco (*Junco hyemalis*): genomic tools for an ecological model system

**DOI:** 10.1186/1471-2164-13-305

**Published:** 2012-07-09

**Authors:** Mark P Peterson, Danielle J Whittaker, Shruthi Ambreth, Suhas Sureshchandra, Aaron Buechlein, Ram Podicheti, Jeong-Hyeon Choi, Zhao Lai, Keithanne Mockatis, John Colbourne, Haixu Tang, Ellen D Ketterson

**Affiliations:** 1 Dept. of Biology, Center for Integrated Study of Animal Behavior, Indiana University, Bloomington, IN, USA; 2 BEACON Center for the Study of Evolution in Action, Michigan State University, East Lansing, MI, USA; 3 Center for Genomics and Bioinformatics, Indiana University, Bloomington, IN, USA; 4 Cancer Center, Department of Biostatistics, Georgia Health Sciences University, Georgia, IN, USA; 5 Greehey Children’s Cancer Research Institute, University of Texas Health Science Center at San Antonio, San Antonio, TX, USA

**Keywords:** Transcriptome, Aves, pyrosequencing, microarray, Junco, 454 titanium cDNA sequencing, single nucleotide polymorphism.

## Abstract

**Background:**

Though genomic-level data are becoming widely available, many of the metazoan species sequenced are laboratory systems whose natural history is not well documented. In contrast, the wide array of species with very well-characterized natural history have, until recently, lacked genomics tools. It is now possible to address significant evolutionary genomics questions by applying high-throughput sequencing to discover the majority of genes for ecologically tractable species, and by subsequently developing microarray platforms from which to investigate gene regulatory networks that function in natural systems. We used GS-FLX Titanium Sequencing (Roche/454-Sequencing) of two normalized libraries of pooled RNA samples to characterize a transcriptome of the dark-eyed junco (*Junco hyemalis*), a North American sparrow that is a classically studied species in the fields of photoperiodism, speciation, and hormone-mediated behavior.

**Results:**

From a broad pool of RNA sampled from tissues throughout the body of a male and a female junco, we sequenced a total of 434 million nucleotides from 1.17 million reads that were assembled *de novo* into 31,379 putative transcripts representing 22,765 gene sets covering 35.8 million nucleotides with 12-fold average depth of coverage. Annotation of roughly half of the putative genes was accomplished using sequence similarity, and expression was confirmed for the majority with a preliminary microarray analysis. Of 716 core bilaterian genes, 646 (90 %) were recovered within our characterized gene set. Gene Ontology, orthoDB orthology groups, and KEGG Pathway annotation provide further functional information about the sequences, and 25,781 potential SNPs were identified.

**Conclusions:**

The extensive sequence information returned by this effort adds to the growing store of genomic data on diverse species. The extent of coverage and annotation achieved and confirmation of expression, show that transcriptome sequencing provides useful information for ecological model systems that have historically lacked genomic tools. The junco-specific microarray developed here is allowing investigations of gene expression responses to environmental and hormonal manipulations – extending the historic work on natural history and hormone-mediated phenotypes in this system.

## Background

Studies of natural populations lie at the core of understanding the evolution of complex, ecologically relevant phenotypes. High-throughput approaches to the study of gene functions have accelerated discoveries of the genetic underpinnings of many traits in model organisms, but until recently organisms with well-understood ecology typically lacked sophisticated genomic tools.

Model laboratory systems have contributed enormously to the understanding of genetics, gene expression, and the functional interactions of genes; however, the ecological relevance of these findings must also be studied within outbred populations responding to natural environmental challenges. By combining the power of natural systems with similar sets of genomics tools developed in laboratory systems, a deeper understanding of the molecular basis of adaptive traits and the mechanisms of biodiversity is achieved in nature. For example, the repeated loss of armor in Alaskan stickleback populations is due to a parallel regulatory mutation [1], and the divergence of cichlid visual systems appears to be driven by changes in expression of opsins, rather than structural changes [2]. Neither of these findings would have been possible without combining the extensive genomic and natural history information available in these systems.

The introduction of high-throughput sequencing technologies has led to significant declines in the time and cost required to generate genomic tools for functional studies. For example, 347 new genomes were published in 2010 alone (NCBI Genome Database, accessed May 2011). However, these genomes are still dominated by invertebrates and prokaryotes. Only 6 of the 347 sequenced genomes were of vertebrates, reflecting the high cost of sequencing large and complex vertebrate genomes. In contrast, transcriptome sequencing – the sequence of all transcripts present in a single cell type, tissue type, or entire organism under defined conditions – has emerged as a cost-effective means of rapidly acquiring functional sequence information for non-model systems [3]. Comprehensive transcriptomes have recently been characterized for several well-studied natural animal species including the Glanville fritillary butterfly [4], staghorn coral [5], horned beetle [6], garter snake [7], great tit [8], and Asian tiger mosquito [9]. These transcriptomes provide species-specific genomic information needed to employ genomic approaches in natural systems where the tools were previously lacking, but without the prohibitive costs and time required for sequencing of a full genome. For example, species-specific microarrays can be developed from transcriptome sequence to assess gene expression in natural populations [6], an approach we employ here. The acquisition of genomic information regarding expressed sequences is a rapid method for identification of meaningful genetic divergence between species (e.g. [10]) and the early gene-expression divergence [11] that is thought to play a major role in speciation [12,13]. Here, we seek to add to this growing store of genomic information.

The dark-eyed junco (*Junco hyemalis*) is a classic avian system that has been extensively studied for more than a century and will be made even more useful with functional genomics. This seasonally-breeding North American sparrow was the first vertebrate animal in which the effect of photoperiod on seasonality was demonstrated [14,15], and its behavior, ecology, and physiology have been extensively studied [16], as has its tendency to diverge phenotypically and genetically across its geographic range [17,18]. One population has been monitored for 30 years and has allowed for assessment of temporal and individual variation in hormone levels, parental behavior, extra-pair mating, and breeding phenology [19,20]. This population has also emerged as a model for phenotypic engineering [21-23]: over 20 studies involving the manipulation of the hormonal phenotypes of free-living individuals have made it possible to relate hormonal variation to variation in phenotype (e.g., [24,25]).

The addition of transcriptome information will allow for deeper understanding of the mechanisms behind variation in behavior and physiology as well as how natural selection acts on that variation. Further, the junco offers unique opportunities to study rapid evolutionary divergence at the level of populations. The dark-eyed junco consists of five morphologically distinct subspecific groups that are thought to have diverged over the past 10,000 years [18]. In addition, approximately 30 years ago a population of juncos colonized the city of San Diego and has undergone rapid phenotypic divergence from its ancestral population in physiology and behavior [26-31]. Previous attempts to reveal the phylogenetic relationships among the groups of juncos have been frustrated by the lack of genetic differentiation owing to recent divergence and a paucity of genetic markers [18]. Genomic-level information will open new lines of research in this natural system including allowing gene-expression analysis, targeted re-sequencing, and identification of genes recently under selection, each of which will allow greater insight into the evolution and mechanisms of hormone-mediated phenotypes and natural breeding biology.

We sequenced a transcriptome of this ecologically and evolutionarily well-characterized species, returning substantial sequence diversity and expression information from a relatively shallow sequence coverage depth of long reads. In this report, we describe our approach and demonstrate the utility of these data in defining genes, identifying potential sequence variants, and confirming expression with a custom microarray.

## Results and Discussion

### Sampling and sequencing

RNA was extracted from 14 tissues (see Methods for full list) from one male and one female adult dark-eyed junco (*Junco hyemalis*) and prepared into normalized sequencing libraries (see Methods for details). Two pools, one from each individual, were sequenced in parallel using GS-FLX Titanium pyrosequencing (Roche/454 Sequencing), yielding 1.17 million reads totaling 434 million nucleotides with a mean length of 372 base pairs (bp) after adaptor trimming. The quality-filtered (cleaned) reads have been deposited in the Sequence Reads Archive (SRA) Database under accession numbers SRX144177.1 and SRX144176.3.

Sampling decisions are a critical question for transcriptome projects as the genes recovered are directly related to the tissues, individuals, and states that are chosen for inclusion. Research in the junco has historically focused on adults and their breeding behavior. The use of only two adult individuals limits the identification of some classes of genes, such as the developmental genes expressed in growing young; however, the decision also improves our ability to confidently assemble sequences by reducing concerns about integrating allelic variation. Future interest in other conditions, or in the identification of more extensive polymorphic markers for comparing populations, will be able to use this assembly as a reference to improve and focus effort on the condition of interest. In other systems, the relative importance of these questions have led to some projects sequencing only developing individuals [32], across multiple ages (e.g. [6]), across multiple eco-types (e.g. [7]), only specific tissues(e.g. [33,34]), or simply more individuals (e.g. [8]). In the junco system, this initial approach provides, in our opinion, the most widely valuable research tools for currently anticipated applications and the strongest base from which to launch future projects.

### Assembly

Sequencing reads were assembled using GS *de novo* Assembler (NEWBLER v2.3; Roche), resulting in 40,564 contigs assembled from 828,612 reads (71% of total, 83% of cleaned reads) covering 35.8 million bases of sequence with an average length of 884 bp and 12-fold average coverage with 166,177 singletons remaining unassembled (Table 1). The assembled contigs have been deposited in the NCBI Transcriptome Shotgun Assembly (TSA) Database under accession numbers JV157086-JV188856. This assembly is similar to other *de novo* transcriptome assemblies, which have been characterized by 40,000 to 50,000 contigs with 63% to 90% of reads assembled [4-8,34]. Variation between individuals and alleles can artificially break contigs, as can alternative splicing [7], so further assembly was required to more accurately estimate the number of unique genes in this transcriptome.

**Table 1:** Sequencing and assembly statistics

	**Number**	**Length**	**Average Length**
Reads	1.17 million	434 million	372
Contigs	40,564	35.8 million	884
Singletons	166,177	57.1 million	344
Isotigs	31,739	NA	1,248
Isogroups	22,765	NA	NA

Sequencing reads were assembled into contigs which were further grouped into isotigs and isogroups (see text for details).

**Figure 1:**
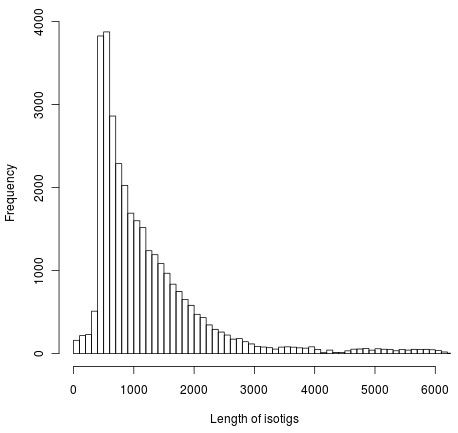
**Distribution of isotig lengths.** The frequency of isotigs of various lengths. Note that isotigs longer than 6,000 bases are omitted from the figure for clarity. The frequency continues to decline for greater lengths.

We further combined contigs into groups based on shared broken reads in the initial assembly following manufacturer's directions (Genome Sequencer FLX System Software Manual, version 2.3, Roche; see Methods for more detail). Briefly, many contigs are broken apart by the assembly software due to variability that can be induced by gene duplications, splice variants or even allelic variation [7]. The GS mapper assembly software stores information about these breaks and then pools contigs that shared broken reads into clusters called isogroups; the contigs within a cluster that are joined by broken reads are called isotigs. In total, the junco assembly yielded 31,739 isotigs (average length of 1248 bases; Figure 1) in 22,765 isogroups (only 4,288 isogroups contain multiple isotigs).

Each isogroup likely represents a gene, while unique isotigs typically represent alternative splice forms; however, the isotigs may also represent divergent alleles, or members of a gene family [7]. The current depth of sequencing precludes an accurate distinction between these possibilities, but future re-sequencing projects and gene expression studies could potentially distinguish between splice variants and recent duplicates. The Ensembl release 63 [35] for the zebra finch (*Taeniopygia guttata;* tae.Gut3.2.4), accessed via BioMart [36,37] contains 19,484 predicted or sequenced genes for the zebra finch, suggesting that our assembly of 22,765 isogroups may be incomplete (multiple isogroups represent a single true gene) or that there are more expressed regions in bird genomes than predicted by current zebra finch sequencing, annotation, and gene models.

### Reference assembly

In order to confirm the validity of our sequences and to test the *de novo* assembly against a reference assembly, we also assembled the junco transcriptome using GMAP [38] with standard parameters against the closest available draft genome assembly: the zebra finch the first passerine genome sequencing project [39], and a species that diverged from juncos approximately 25 Ma ago [40]. Of the 1,180,500 reads from the junco transcriptome used for this assembly, 1,031,427 (87%) had a significant alignment to the zebra finch genome identified by the software, with an average of 92.9% identity. However, due to the limitation imposed by introns, the lengths of the assembled regions from the reference assembly are substantially shorter (331 bp vs 872 bp), and split into many more groups, than our *de novo* assembly (see Additional file 1 for further information on statistics and approach). For this reason, we chose to use the *de novo* assembly for all further analyses.

### Annotation

The isotigs and singletons were queried against the NCBI non-redundant protein database[41,42] using BlastX to identify homologous, annotated genes. From this search, 17,884 of the isotigs and 15,871 of the singletons (49% and 11% of total respectively) returned a significant homolog (e value < 10^-5^; Table 2; should refer to Additional file 2 for full annotation from all approaches). The proportion of assignment for isotigs is slightly higher than that of other *de novo* transcriptomes (range 23% - 35%), possibly because our isotigs are longer than the contigs used for annotation in other transcriptome annotations (1,248 bp vs 197 to 871 bp) [4-8,34]. Isotigs covered an average of 56.4% of the protein to which they aligned; singletons covered an average of only 18.4% of the protein to which they aligned, consistent with their shorter length. Isotigs were, on average, 82.4% identical across the full length of their alignment, while singletons were 79.4% identical.

**Table 2:** Number of isogroups and singletons annotated by different approaches, confirmed expressed, and containing SNPs

**Assignment**	**Isogroup**	**Singleton**
Total Assembly	22,765	166,177
NCBI nr-protein	10,276	15,871
Ensembl - ZF	9,863	14,019
Gene Ontology	9,120	737
KEGG	3,827	3,984
OrthoDB	10,062	14,019
Expressed	16,781	16,096
SNP present	6,992	NA

Sequences were annotated by sequence similarity to multiple databases, yielding the above number of annotations.

Because isogroups represent the full genetic unit, we sought to combine the isotigs into a single annotation for each isogroup, resulting in an annotation for 48% of isogroups (11,015 of 22,765). For nearly all annotated isogroups (10,276; 93%), only a single annotation was identified for all of its member isotigs. For a subset of isogroups (716; 7%), multiple annotations were identified but these were confirmed synonymous by literature or alignment searches to confidently assign an isogroup annotation (see Methods for details). Some isogroups (23; <1%) could not be reduced to a single annotation and might represent errors in assembly or transcripts with no currently identifiable homology. Our ability to assign a single annotation to most (99%) of annotated isogroups suggests that our assembly accurately characterizes many of the genes of the junco.

Of the 7,918 unique annotations identified among the isogroups reduced to a single annotation, 6,722 (85%) are represented by a single isogroup; the remaining 1,196 are represented by 4,268 isogroups (average of 3.57 isogroups per annotation). Multiple isogroups assigned to a single annotation suggests that some true genes in our dataset may be incompletely assembled, or represent recently diverged gene duplicates. For example, 6 isogroups are annotated as “nebulin,” which is a conserved, single-copy gene in vertebrates [43,44], coding for an actin binding protein with multiple isoforms [45] that can complicate transcriptome assembly. The human version of nebulin has 6,669 amino acids and contains 183 exons at least 43 of which are alternatively spliced [46]. Additionally, some annotations are not for specific gene products: there are 1,353 isogroups assigned to nine annotations named some variant of “unnamed” or “hypothetical” proteins. The presence of multiple isogroups annotated as a single gene suggests that further assembly, or additional sequencing, may be necessary to complete the assembly of some large or complex genes.

### Functional Annotation

Gene Ontology (GO) is a hierarchical description of gene function that classifies genes based on known or predicted function in model organisms [47]. The use of GO terms allows a broader assessment of our annotation and permits the assignment of functional roles to individual genes. There are limitations to assigning GO terms by sequence similarity alone, which can result in over-assignment of GO terms to genes that have functionally diverged [48-50]. Consequently, we are not placing high levels of confidence in any particular GO assignment, but rather we are investigating the large-scale patterns revealed by these functional annotations. While there are still some concerns with this approach, preliminary microarray results reveal meaningful and expected GO terms (Peterson, Rosvall, Tang and Ketterson, unpublished data), suggesting that our functional annotation is sufficient for broad assessments with the caveat that confidence in any given assignment should be limited.

We assigned GO terms, based on sequence similarity, using Blast2GO [51] and set a strict threshold criterion of e < 10^-15^ against the NCBI non-redundant protein database [41,42]. This process provided at least one GO term for 83% of annotated isogroups (9,120) and 5% of annotated singletons (737) at the “inferred from sequence similarity” level of evidence [47]. This reduced ability to functionally classify singletons is likely due to their shorter length (mean 344 bp vs. 1,248 bp), which reduces the length and quality of sequence alignments that are possible, especially considering the stringent criterion employed. We identified a total of 65,008 GO term annotations representing 3,515 unique GO terms distributed throughout the GO graph (should refer to Additional file 2 for full annotations).

### Pathway annotation

We additionally annotated the junco transcriptome sequences by mapping to the Kyoto Encyclopedia of Genes and Genomes (KEGG) pathway annotation [52-54]. Briefly, assembled isotigs and singletons were aligned to protein sequences from the Ref-Seq databases [41,42] for zebra finch, chicken, mouse, and human using tBlastX, resulting in 6,269 isotigs (from 3,827 isogroups) and 3,984 singletons being assigned across all 234 unique pathways available for annotation (should refer to Additional file 2). These pathways can, in the future, be targeted for manipulation or sequencing. In addition, gene expression studies will likely use this approach to analyze changes to whole pathways, an approach which may be more sensitive and interpretable than focusing on single genes [55], to identify small changes in expression level with phenotypic consequences.

### Orthology group assignment

In addition to our other annotations, we assigned the junco sequences to OrthoDB [56] orthology groups. We aligned junco sequences to the Ensembl protein database for zebra finch [35] with BlastX with a criteria of e < 10^-10^, and assigned the top alignment (by bit score) to each isotig and singleton. We then determined whether all isotigs in an isogroup were assigned to the same protein. This returned a single annotation for 9,863 isogroups (43%), and multiple annotations for 199 isogroups that we excluded from further portions of this analysis. In addition, 14,019 singletons (8%) were assigned to an Ensembl protein. Among the isogroups, 6,542 annotations were assigned to only a single isogroup, and an additional 1,442 were assigned to multiple isogroups. Including the singletons returned 2,830 additional unique annotations. We then used the OrthoDB database [56] to assign each junco sequence as the orthology group that the corresponding zebra finch protein. This process resulted in assignment to 9,633 of the 12,557 orthology groups previously identified in zebra finch [56]. These data will allow future studies to focus on the divergence of orthologous gene families between junco and closely related species and may aid in the identification of recently duplicated genes in the junco.

### Completeness

In order to assess the completeness of this transcriptome based on our sequencing efforts, we searched for significant sequence alignments for the assembled isotigs and singletons against the eukaryotic clusters of orthologous group (KOG) database [57,58]. Specifically, we searched for a set of 716 genes identified to be present as single copies in all bilaterians (an animal clade, including deuterostomes and proterostomes, that diverged at least 555 Ma ago) studied to date [59]. This reciprocal Blast analysis identified homologs for 646 core genes (90.2%) in our dataset (should refer to Additional file 2 for full annotation). This high level of coverage is slightly lower (but still comparable) to that found for large-scale genome sequences, which generally identify 99% of these core genes [59]. The best junco alignment for each KOG group aligned with a mean of 82.4% identity and covered an average of 69.5% (301.8 amino acids) of the aligned protein. This alignment coverage is likely artificially reduced by the fact that only one isotig (rather than a full isogroup) was counted for this length, meaning that any alternative splicing (or allelic variation) could result in a low reported coverage. Other transcriptome projects have not, to our knowledge, used this analysis. These results provide strong support that the junco transcriptome has been sequenced to sufficient depth and from a sufficiently diverse pool of sampled RNA to uncover most of the expected genes.

### Confirmation of expression

To confirm that our putative transcripts are expressed, we constructed a custom 12-plex microarray (Roche NimbleGen, Inc., Madison, WI; see Methods for details) and conducted an expression experiment involving four tissues (ventromedial telencephalon, hypothalamus, liver, and pectoralis muscle) in male and female juncos. The full results of this experiment will be described elsewhere; this initial analysis focuses simply on confirming the expression of genes in the tissues of wild-caught individuals. We confirmed the expression of 23,914 (71.3%) of the contigs (representing 16,871 (74%) isogroups) and 16,096 (46.8%) of the singletons on the array in at least one sex-tissue combination (should refer to Additional file 2 for list). The isogroups and singletons that did not show expression in these analyzed tissues may be tissue/condition-specific genes that are unexpressed in our sampled tissues, or could represent spurious sequences that do not accurately represent expressed transcripts. On the whole, this result suggests that our sequenced genes are largely accurate reflections of expressed genes in the junco.

### Sequence variants

Allelic variants, such as single nucleotide polymorphisms (SNPs), are powerful tools for population genetic analysis and identification of population structure. The junco system currently only has nine microsatellite markers, which have been used extensively for paternity analysis in one population [20]. In addition, 243 SNPs were identified across multiple junco species, and this number was sufficient to identify species, but not subspecies, divergence [60]. The addition of more genetic markers will greatly improve the ability to do large population comparisons, including further refining the current junco subspecies phylogeny [18], and identifying variants that may play a role in the current rapid divergence of the genus *Junco*. To this end, we have conservatively identified 25,781 unique potential SNPs in 6,992 isogroups of this transcriptome (see Additional file 3 for full list and methods for details) for an average rate of 0.72 potential variants per 1000 basepairs of assembled sequence. While this rate of sequence variant identification is low compared to transcriptomes sequencing a greater number of individuals [7], these potential sequence markers will provide a strong starting point for future studies on population genetics and divergence in the genus *Junco*.

## Conclusions

We have successfully sequenced a transcriptome of an ecological model songbird, the dark-eyed junco, using pyrosequencing and de novo assembly. Through our assembly process, we identified 22,765 putative genes – half of which have been annotated and three-quarters of which were validated by microarray investigation. These genes, including over 25,000 potential sequence variants, will immediately begin to inform the study of the junco and will provide a valuable resource for the study of many songbird species. Based on the identification of putatively conserved genes, we have demonstrated 90% coverage.

The acquisition of these sequence data adds genomic resources to another non-model system. By combining these data (and the tools they produce) with the historical study of a natural population, a solid foundation has been laid to advance the study of ecology, evolution, and behavior. We have already begun to exploit these novel tools in gene expression studies, showing in this approach that the majority of our sequenced genes are expressed under at least some natural conditions. Further research will extend these tools to deeper study of the transcriptomic responses of juncos to environmental stimuli, as well as assist in guiding traditional sequencing projects.

## Methods

### Tissue collection and RNA extraction

We collected tissues from two adult dark-eyed juncos, one male and one female, for this transcriptome. Both individuals had been held in captivity from 2005 when they were captured as six-day old nestlings in the wild near Mountain Lake Biological Station in Giles County, VA (37° 22′ 31″N, 80° 31′ 24″W), and transported to Bloomington, IN (39° 09′ 02″N, 86° 23′ 46″W). Individuals were hand-reared on a standard diet and were not manipulated in any experiment prior to inclusion in the transcriptome. For six months prior to euthanasia, we housed the birds individually, but neither visually nor acoustically isolated, on a photoperiod of 16:8 (hours light: dark) to induce gonadal growth and to ensure that we sequenced genes expressed during long photoperiods.

Within 20 min of euthanization by an overdose of isoflurane, all tissues were removed from the individual, ground in TRIzol® (Invitrogen Life Sciences, Carlsbad, CA), and stored at −80 °C. We collected the following organs: whole brain, gonad, liver, pectoralis muscle, syrinx, beak, eye, gizzard, heart, kidney, lung, preen gland, skin, and tongue. These tissues were chosen to represent a wide swath of potential gene expression, while explicitly avoiding the tissues (e.g., stomach, spleen) that were most likely to contain other species. This procedure conformed to all animal care regulations and was approved by the Bloomington Institutional Animal Care and Use Committee at Indiana University (Protocol #09-037). We extracted RNA from each tissue separately for each individual following the TriReagent manufacturer's protocol (Invitrogen Life Sciences; [61]). Total RNA was resuspended in water, and we confirmed concentration and quality with a Bioanalyzer nanochip (Agilent Technologies, Waldbronn, Germany).

### Library preparation and sequencing

Total RNA from each individual was quantified by fluorimetry (Quant-iT™ RiboGreen®, Invitrogen) and prepared into equimolar pools of 800 ng, creating whole-body male and female pools. Sequencing libraries optimized for Roche/454 Titanium sequencing were prepared using IU CGB customized protocols as previously described in [7] modified from [5]. Briefly, cDNA was synthesized in a fashion similar to the Clontech™SMART system by PCR amplifying each RNA pool using primers optimized for 454 sequencing. The resulting double-stranded cDNAs was then normailized by treatment with duplex-specific nuclease to reduce representation of highly abundant transcripts. Male and female libraries were separately titrated by enrichment and prepared for sequencing by emulsion PCR, each on one region of a two-region GS-FLX Titanium PicoTitre^TM^ plate. The reads were cleaned of all adaptor/primer and polyA sequence by a program developed in-house at the CGB, Indiana University (http://sourceforge.net/projects/estclean/ website). After cleaning, sequences ≤30 bp were removed from the dataset.

### Assembly

Reads from the male and female pools were combined to increase the accuracy and completeness of assembly. We assembled these pooled reads using NEWBLER (v2.3; Roche/454 Sequencing) with the default parameters (40 bp overlap; 90% identity) resulting in 40,564 contigs (and 166,177 remaining singletons), which were further assembled into isogroups. Schwartz et al. [7] previously referred to this approach as graph-clustering and contig-graphs, as it graphically combines clusters of contigs that appear to be transcribed together (Genome Sequencer FLX System Software Manual, version 2.3, October 2009). An isogroup is composed of contigs that were split during the initial assembly because some of the reads overlapped multiple, independent contigs. NEWBLER reports information about reads that were broken between contigs during assembly, and clusters the component contigs into a single isogroup representing a putative gene. Once an isogroup is formed, all potential paths through the cluster are traversed and those paths that are supported by broken reads are reported as isotigs – that is, putative transcripts. Isogroups can either represent alternatively spliced genes (with contigs indicating exons, and isotigs representing splice forms), or sets of recently duplicated genes (with contigs representing regions of divergence since duplication, and isotigs representing the divergent genes) either as gene families or multiple alleles of the same gene [7].

### Annotation

After determining that the *de novo* assembly was superior to the reference assembly, we used isotigs and singletons as a query against the NCBI non-redundant protein database (Accessed in October 2010) using BlastX sequence alignment with a threshold e-value of 10^-5^. We identified the top match for each isotig or singleton by bit-score, and the corresponding gene information was assigned to the junco sequence. Because a number of genes are referred to by multiple names and abbreviations, we manually curated those isogroups that contained multiple unique annotations to identify a single annotation for each isogroup. For the majority of multiply annotated isogroups (637), this involved simply collapsing multiple synonyms, but 102 isogroups contained annotations that could not be readily collapsed. The isotigs from these isogroups were queried against the zebra finch UniProt database using BlastX with strict threshold criterion (e value < 10^-10^) to reduce spurious matches from divergent taxa. The top five sequence alignments (by bit score) for each isotig in an isogroup were compared and if a single gene appeared in all isotigs and was represented in the original annotation, it was assigned as the isogroup annotation. For cases in which two or more gene annotations were identified in all isotigs from an isogroup, the annotation with the highest cumulative bit score was assigned as the isogroup annotation if it matched an original annotation. This approach left 23 isogroups with multiple annotations; 7 did not match against anything in the zebra finch UniProt database, and 16 matched against genes other than those originally annotated and could not be fully collapsed – these isogroups were omitted from further isogroup analyses.

### Additional Annotation

We also used isotigs and singletons to query several databases designed to provided additional information on the function, pathway, orthology group, and completeness of the assembled sequences. Table 3 details the databases and search parameters utilized for these annotations. The top alignment match from each search was assigned to the corresponding isogroup or singleton.

**Table 3:** Additional annotation approach details

**Approach**	**Database**	**Blast Flavor**	**E-value**	**References**	**Notes**
Functional Annotation	NCBI- NR	Blast2GO	10^-15^	[41,42,47,51]	
Pathway Annotation	KEGG	tBlastX	10^-5^	[52-54]	Limited to zebra finch, chicken, and mouse.
Orthologous Group	OrthoDB, Ensembl	BlastX	10^-10^	[35-37,56]	Limited to zebra finch. Matched Ensembl hits to OrthoDB assignments

To obtain more detailed information about the function of the sequenced genes, searches beyond simple non-redundant proteins were conducted.

### Completeness

We used the predicted open reading frames from the assembled junco sequences (isotigs and singletons) to search for a set of 716 conserved bilaterian genes [59] retrieved from the KOG protein database [57,58]. Reciprocal BlastP was performed with an e-value threshold of e < 10^-5^. Junco sequences were identified as the homolog of a KOG protein if, and only if, they were reciprocal best alignments. The best junco alignment for each KOG group was then selected by bit score and alignment statistics were reported.

### Confirmation of expression

A custom microarray was designed from the sequence of the junco transcriptome. For 33,545 contigs, three unique probes are present on the array, while another 61 contigs are represented by two probes and 65 contigs are represented by one probe, accounting for 100,822 probes on the array. An additional 34,365 probes were selected from the remaining singletons (one probe per chosen singleton). The array also contains control probes and 2,604 random probes designed to reflect the genome nucleotide composition by Markov modeling to experimentally determine the appropriate thresholds that measure significant hybridization signals over the background. Thus, each sub-array consists of over 137,000 long-oligonucleotide (60 bp) probes, and 12 such sub-arrays are placed on each glass slide (Roche NimbleGen Inc., Madison, WI). The microarray platform is deposited at NCBI Gene Expression Omnibus (GEO; accession number GPL14995).

We collected adult dark-eyed juncos from breeding grounds near Mountain Lake Biological Station (Pembroke, VA) in mist-nets between May 7 and 14, 2010 and held them individually in a semi-naturalistic outdoor aviary where they were neither acoustically nor visually isolated from other juncos, as part of a larger experiment. On June 9 and 10 individuals were euthanized by overdose of isoflurane. Tissues, including whole brains, were collected rapidly and stored on powdered dry ice within 20 min post-mortem to ensure negligible RNA degradation [62]. Brains were dissected into 14 distinct regions using anatomical landmarks, following previously established methods [63] based on the zebra finch brain atlas. These brain regions included the hypothalamus and the ventral medial telencephalon (VmT), which primarily consists of the nucleus taeniae, the avian homologue of the medial amygdala [64-66].

RNA from VmT, hypothalamus, liver, and pectoralis was extracted in TRIzol® following manufacturer's directions (Invitrogen, Carlsbad, CA). The microarray protocol follows previously published methods [67]. Briefly, total RNA was reverse-transcribed to ss-cDNA in the presence of oligodT primer and SuperScript II reverse transcriptase. This ss-cDNA was then converted to ds-RNA and labeled using CY-labeled random nonmer primer (either Cy3 or Cy5) and Klenow fragment (following NimbleGen labeling protocols). We then hybridized 4 g of each of two labeled samples (one Cy3, one Cy5) to each sub-array and followed manufacturer's directions for post-hybridization washing and scanning (Roche NimbleGen, Inc., Madison, WI). Imaging was accomplished by Axon GenePix 4200A scanner (Molecular Devices, Sunnyvale CA) with GenePix 6.0 software and data were extracted with NimbleScan 2.4 (Roche NimbleGen, Inc., Madison WI). Raw microarray data were processed with the limma package [68] in R version 2.9.0 [69] to normalize expression scores.

To determine if a gene was expressed, we calculated the 97.5% quantile for expression score of random probes in each individual as the cutoff for calling expression. Thus, for each individual, a called expression is significant at a p-value of 0.025. For each contig, we tested the median probe value against this threshold, and for singletons we used the single expression value. Because our design employed biological replicates, we called a contig or singleton expressed only if at least three of the six individuals in a group were called as expressed, thus reducing the p-value further to 0.0006 (the probability of obtaining at least three of six individuals called for expression of a random probe). From this, we determined whether or not a gene had expression support in any of our tissues-sex pairings, and which genes were restricted to expression in one sex.

### Sequence Variants

To identify sequence variants, we aligned cleaned sequence reads to the assembled transcriptome using Blastn with a threshold of e < 10^-5^ and requiring 95% identity and alignment of all but the last five bases on each end of the read. The first and last five bases on each end of the read were trimmed before analysis (if they aligned) to prevent inaccurate SNP calls near the edges of reads. Reads that were assembled to multiple isogroups were omitted from further analysis. The aligned reads at each sequence position (base) were compared to the consensus sequence (the assembly).

Those positions that contained a minor allele represented with a frequency greater than 20%, and supported by at least three reads, were identified as potential SNPs. Alleles representing gaps were not included in this dataset because insertions and deletions are the most common form of 454 sequencing error [70] and generally cause frameshift mutations in coding sequence (such as cDNA sequencing) and are therefore less likely to be true sequence variants. Sequencing errors for GS-FLX Titanium pyrosequencing (Roche/454 Sequencing) mismatches at a rate of 0.022% [70], suggesting that we should only expect 0.37 false SNPs (due to sequencing error) to be called under these stringent criteria (binomial distribution of at least three substitution errors at the same site with depth of coverage eight = 1.055 × 10^-8^; times 35.8 million sites), providing strong support that any identified sequence variants are very likely to represent true SNP variation in these two individuals.

Because SNPs were identified in isotigs, there is the potential to identify the same SNP in more than one isotig of the same isogroup. To address this concern, we report the number of confidently unique SNPs along with the total number identified. Called SNPs that had the same major and minor allele were restricted to only those from the isogroup with the greatest number of that sequence variant. For example, in isogroup00018, 250 isotigs all had calls for SNPs with G as the major allele, and T as the minor allele at position 551 (likely because they all share the same first contig). Only the call for this polymorphism from a single isotig was included in the report of unique SNPs. This approach was conservative, as we likely omitted SNPs that were truly unique as well.

## Competing interests

The authors declare no competing interests.

## Authors’ contributions

MP coordinated the sequencing and annotation projects, led the microarrary experiment, combined the data from all analyses, manually curated annotations, performed orthologous group annotation and microarray analysis, ensured accuracy of all aspects of analysis, and drafted the manuscript. DW coordinated biological interpretations, assisted with animal care and contributed substantially to the intellectual content of the manuscript. SA conducted and analyzed the reference assembly and provided feedback on annotation approaches. SS annotated sequences for pathway and completeness analysis and produced alignments for difficult to assign isogroups. AB performed annotations, and provided feedback on many stages of bioinformatics. RP conducted the analysis of sequence variants. JHC led annotation pipeline construction and oversaw all annotation as well as performed the preliminary limma analysis for microarray experiments. ZL oversaw RNA sample and library preparation and led initial *de novo* assembly efforts. KM designed and directed transcriptome sequencing, submitted sequencing data and edited the manuscript. JKC oversaw all genomics aspects of the analysis, identified appropriate annotation methods for pathway and completeness analysis as well as performing initial expression analysis, submitted microarray data, and contributing substantially to the manuscript. HT led all bioinformatic aspects of the analysis, coordinated the multiple levels of analysis and ensured statistical rigor. EK led all biological aspects of the analysis, including coordinating animal use and biological interpretations as well as contributing substantially to the intellectual content of the manuscript. All authors have contributed to the interpretation of results and read and approved the final manuscript.

## Supplementary Material

Additional file 1 Contains statistics and a brief description of our attempt to develop a reference assembly using the zebra finch genome [71-75]. Click here for file

Additional file 2 List of isogroups and singletons (rows) with their corresponding annotations (from various sources of evidence) and expression support (columns). Click here for file

Additional file 3 SNPs identified in the assembled transcriptome, including both unique and redundant (i.e., the same SNP call in multiple isotigs of the same isogroup) SNP calls. Click here for file
