# Supplementary material for: De novo transcriptome sequencing in a songbird, the dark-eyed junco (Junco hyemalis): genomic tools for an ecological model system
Source: BMC Genomics. 2012 Jul 9;13:305. doi: 10.1186/1471-2164-13-305 (PMC3476391; doi:10.1186/1471-2164-13-305)
Supplement: Additional file 1 — Contains statistics and a brief description of our attempt to develop a reference assembly using the zebra finch genome [71-75]. [file 1471-2164-13-305-S1.pdf]

Reference Assembly

In order to confirm the validity of our sequences and to test the *de novo* assembly against a reference assembly, we also assembled the junco transcriptome using GMAP [1] with standard parameters against the closest available draft genome assembly: the zebra finch the first passerine genome sequencing project [2], and a species that diverged from juncos approximately 25 million years ago [3]. Briefly, each read was independently aligned to the genome at each location with a match meeting the program's 24-mer criteria to ensure that large intron interruptions did not artificially reduce our sequence alignment rates.

Of the 1,180,500 reads from the junco transcriptome used for this assembly, 1,031,427 (87%) had a significant alignment to the zebra finch genome identified by the software, with an average of 92.9% identity. The high proportion of matches strongly suggests that the sequencing reads from the junco transcriptome are high quality. The high identity supports the relatively close relationship between junco and zebra finch.

In addition, we identified the overlap of locations on the zebra finch genome that had been aligned to a junco read in order to identify regions of the genome that were transcribed in un-interrupted fashion (i.e., exons). Junco sequences aligned in 116,201 regions, representing 26,651,558 unique basepairs, in the zebra finch genome (average length of 331 basepairs, 10.5-fold average depth of coverage). This length is within the empirically identified range of the length of exons in several taxa (150 to 370 bp; [4]), as expected if our sequence reads were from transcribed RNA rather than genomic DNA. The Ensembl release 63 of gene models for the zebra finch [5] contains a total predicted transcribed length of 24,471,883 base pairs. The fact that there are more matches than the predicted length of genes indicates that we have extensive transcriptome coverage.

Many of the reads that mapped to the zebra finch genome aligned to multiple locations (585,709; 56.78% of mapped reads); however, only 8,866 (1.51% of multiply mapped reads) matched to locations on multiple chromosomes, far less than expected by chance ($\square^2$, d.f. = 1, $p < 0.001$). This finding strongly suggests that the junco 454 reads were split by introns located in the genomic DNA, which is to be expected in sequencing reads that contain only exons and not genomic DNA. Due to the limitation imposed by introns, the lengths of the assembled regions from the reference assembly are substantially shorter, and split into many more groups, than our *de novo* assembly. For this reason, we chose to use the *de novo* assembly for all further analyses.

References

1. Wu TD, Watanabe CK: **GMAP: a genomic mapping and alignment program for mRNA and EST sequences**. *Bioinformatics* 2005, **21**:1859-1875.
2. Warren WC, Clayton DF, Ellegren H, Arnold AP, Hillier LW, Kuenstner A, Searle S, White S, Vilella AJ, Fairley S, Heger A, Kong L, Ponting CP, Jarvis ED, Mello CV, Minx P, Lovell P, Velho TAF, Ferris M, Balakrishnan CN, Sinha S, Blatti C, London SE, Li Y, Lin YC, George J, Sweedler J, Southey B, Gunaratne P, Watson M, Nam K, Backstroem N, Smeds L, Nabholz B, Itoh Y, Whitney O, Pfenning AR, Howard J, Voelker M, Skinner BM, Griffin DK, Ye L, McLaren WM, Flicek P, Quesada V, Velasco G, Lopez-Otin C, Puente XS, Olender T, Lancet D, Smit AFA, Hubley R, Konkel MK, Walker JA, Batzer MA, Gu W, Pollock DD, Chen L, Cheng Z, Eichler EE, Stapley J, Slate J, Ekblom R, Birkhead T, Burke T, Burt D, Scharff C, Adam I, Richard H, Sultan M, Soldatov A, Lehrach H, Edwards SV, Yang SP, Li XC, Graves T, Fulton L, Nelson J, Chinwalla A, Hou S, Mardis ER, Wilson RK: **The genome of a songbird**. *Nature* 2010, **464**:757-762.
3. Barker F, Cibois A, Schikler P, Feinstein J, Cracraft J: **Phylogeny and diversification of the largest avian radiation** . *Proc Natl Acad Sci U S A* 2004, **101**:11040-11045.
4. Zhu L, Zhang Y, Zhang W, Yang S, Chen JQ, Tian D: **Patterns of exon-intron architecture variation of genes in eukaryotic genomes**. *BMC Genomics* 2009, **10**.
5. Flicek P, Amode MR, Barrell D, Beal K, Brent S, Chen Y, Clapham P, Coates G, Fairley S, Fitzgerald S, Gordon L, Hendrix M, Hourlier T, Johnson N, Kaehaeri A, Keefe D, Keenan S, Kinsella R,

Kokocinski F, Kulesha E, Larsson P, Longden I, McLaren W, Overduin B, Pritchard B, Riat HS, Rios D, Ritchie GRS, Ruffier M, Schuster M, Sobral D, Spudich G, Tang YA, Trevanion S, Vandrovcova J, Vilella AJ, White S, Wilder SP, Zadissa A, Zamora J, Aken BL, Birney E, Cunningham F, Dunham I, Durbin R, Fernandez-Suarez XM, Herrero J, Hubbard TJP, Parker A, Proctor G, Vogel J, Searle SMJ: **Ensembl 2011**. *Nucleic Acids Res* 2011, **39**:D800-D806.
